# 2-[(*E*)-2-(1*H*-Indol-3-yl)ethen­yl]-1-methyl­pyridinium 4-chloro­benzene­sulfonate^1^

**DOI:** 10.1107/S1600536809029547

**Published:** 2009-07-31

**Authors:** Thawanrat Kobkeatthawin, Suchada Chantrapromma, Hoong-Kun Fun

**Affiliations:** a Crystal Materials Research Unit, Department of Chemistry, Faculty of Science, Prince of Songkla University, Hat-Yai, Songkhla 90112, Thailand; b X-ray Crystallography Unit, School of Physics, Universiti Sains Malaysia, 11800 USM, Penang, Malaysia

## Abstract

In the title compound, C_16_H_15_N_2_^+^·C_6_H_4_ClO_3_S^−^, the cation exists in an *E* configuration with respect to the central C=C bond and is approximately planar, with a dihedral angle of 2.95 (5)° between the pyridinium and indole rings. The mean plane of the π-conjugated system of the cation and the benzene ring of the anion are inclined to each other at a dihedral angle of 69.65 (4)°. In the crystal packing, the cations are stacked in an anti­parallel manner along the *a* axis, resulting in a π–π inter­action with a centroid–centroid distance of 3.5889 (7) Å. The anions are linked into a chain along the *a* axis by weak C—H⋯O inter­actions. The cations are linked with the anions into a three-dimensional network by N—H⋯O hydrogen bonds and weak C—H⋯O inter­actions. There are also short O⋯Cl [3.1272 (10) Å] and C⋯O [3.1432 (14)–3.3753 (14) Å] contacts. The crystal structure is further stabilized by C—H⋯π inter­actions.

## Related literature

For bond-length data, see: Allen *et al.* (1987 ▶). For background to non-linear optical materials research, see: Ogawa *et al.* (2008 ▶); Weir *et al.* (2003 ▶); Yang *et al.* (2007 ▶). For related structures, see: Chanawanno *et al.* (2008 ▶); Chantrapromma *et al.* (2006 ▶, 2007 ▶, 2008 ▶, 2009 ▶). For the stability of the temperature controller used in the data collection, see: Cosier & Glazer (1986 ▶).
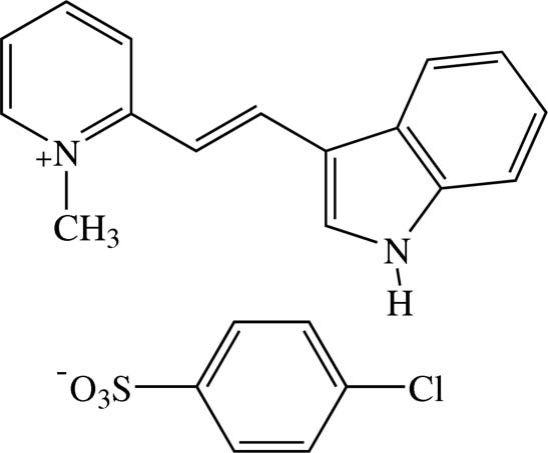


## Experimental

### 

#### Crystal data

C_16_H_15_N_2_^+^·C_6_H_4_ClO_3_S^−^
*M* *_r_* = 426.91
Monoclinic, 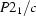

*a* = 7.4891 (1) Å
*b* = 13.1650 (1) Å
*c* = 20.3428 (2) Å
β = 98.801 (1)°
*V* = 1982.06 (4) Å^3^
*Z* = 4
Mo *K*α radiation
μ = 0.33 mm^−1^
*T* = 100 K
0.34 × 0.28 × 0.19 mm

#### Data collection

Bruker APEXII CCD area-detector diffractometer
Absorption correction: multi-scan (**SADABS**; Bruker, 2005 ▶) *T*_min_ = 0.899, *T*_max_ = 0.942
39049 measured reflections
8706 independent reflections
7032 reflections with *I* > 2σ(*I*)
*R*_int_ = 0.034

#### Refinement

*R*[*F* ^2^ > 2σ(*F* ^2^)] = 0.040
*wR*(*F* ^2^) = 0.110
*S* = 1.05
8706 reflections
267 parameters
H atoms treated by a mixture of independent and constrained refinement
Δρ_max_ = 0.88 e Å^−3^
Δρ_min_ = −0.41 e Å^−3^

### 

Data collection: *APEX2* (Bruker, 2005 ▶); cell refinement: *SAINT* (Bruker, 2005 ▶); data reduction: *SAINT*; program(s) used to solve structure: *SHELXTL* (Sheldrick, 2008 ▶); program(s) used to refine structure: *SHELXTL*; molecular graphics: *SHELXTL*; software used to prepare material for publication: *SHELXTL* and *PLATON* (Spek, 2009 ▶).

## Supplementary Material

Crystal structure: contains datablocks global, I. DOI: 10.1107/S1600536809029547/is2441sup1.cif

Structure factors: contains datablocks I. DOI: 10.1107/S1600536809029547/is2441Isup2.hkl

Additional supplementary materials:  crystallographic information; 3D view; checkCIF report

## Figures and Tables

**Table 1:** Hydrogen-bond geometry (Å, °)

*D*—H⋯*A*	*D*—H	H⋯*A*	*D*⋯*A*	*D*—H⋯*A*
N2—H1*N*2⋯O1^i^	0.891 (18)	1.864 (19)	2.7541 (14)	176.2 (18)
C1—H1*A*⋯O3	0.93	2.53	3.2380 (14)	133
C7—H7*A*⋯O2^ii^	0.93	2.59	3.3067 (14)	134
C14—H14*A*⋯O2^iii^	0.93	2.52	3.2605 (14)	137
C16—H16*C*⋯O2^iii^	0.96	2.37	3.2645 (15)	156
C19—H19*A*⋯O1^iv^	0.93	2.30	3.1432 (14)	151
C21—H21*A*⋯O3^iii^	0.93	2.55	3.1885 (14)	127
C4—H4*A*⋯*Cg*3^v^	0.93	2.85	3.5956 (11)	138
C16—H16*A*⋯*Cg*1^vi^	0.96	2.72	3.4622 (12)	134
C16—H16*B*⋯*Cg*3	0.96	2.67	3.5533 (11)	153

Symmetry codes: (i) 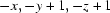; (ii) 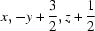; (iii) 
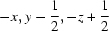
; (iv) 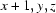; (v) 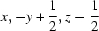; (vi) 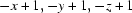. *Cg*1 and *Cg*3 are the centroids of the N2/C8–C9/C10/C15 and C10–C15 rings, respectively.

**Fig. 1.**
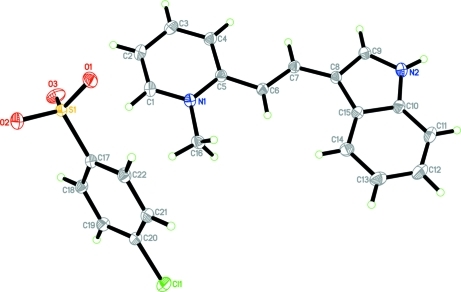
The molecular structure of the title compound, with 50% probability displacement ellipsoids and the atom-numbering scheme.

**Fig. 2.**
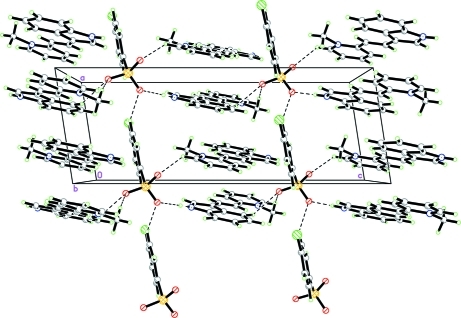
The crystal packing of the title compound viewed down the b axis. Hydrogen bonds are shown as dashed lines.

## supplementary crystallographic information

### Comment

Molecules with extensive conjugated π systems are attractive candidates for non-linear optical (NLO) studies (Ogawa *et al.*, 2008; Weir *et al.*, 2003; Yang *et al.*, 2007). However a molecule with extensive conjugated π systems does not always exhibit second order NLO properties unless the alignment of these molecules is in a noncentrosymmetric space group in the crystal. In our NLO research we have solved a number of crystal structures of pyridinium salt derivatives (Chanawanno *et al.*, 2008; Chantrapromma *et al.*, 2006, 2007, 2008, 2009) which we attempt to examine in details of the relationship between their crystal packings and the NLO properties. We herein report the crystal structure of the title compound (I) which is iso-structure and iso-packing with 2-[(*E*)-2(1*H*-Indol-3-yl)ethenyl]-1-methylpyridinium 4-bromobenzenesulfonate (Chantrapromma *et al.*, 2009).

Figure 1 shows the asymmetric unit of (I) which consists of a C_16_H_15_N_2_^+^ cation and a C_6_H_4_ClO_3_S^-^ anion. The cation exists in the *E* configuration with respect to the C6═C7 double bond [1.3567 (14) Å] and is essentially planar with the dihedral angle between the pyridinium and indole rings being 2.96 (5)° and the torsion angles C4–C5–C6–C7 = -1.21 (17)° and C6–C7–C8–C15 = -176.40 (11)°. The indole ring system is planar with the maximum deviation of 0.014 (1) Å for atom C8. The mean planes through π-conjugated systems of the cation and the anion are inclined to each other with an interplanar angle of 69.65 (4)°. The methyl group is co-planar with the attached N1/C1–C5 ring. The bond lengths in (I) are in normal ranges (Allen *et al.*, 1987) and are comparable with those in related structures (Chanawanno *et al.*, 2008; Chantrapromma *et al.*, 2006, 2007, 2008, 2009).

In the crystal packing (Fig. 2), all O atoms of the sulfonate group are involved in weak C—H···O interactions (Table 1). The arrangement of the cations and anions is interesting (Fig. 2). The cations are stacked in an antiparallel manner along the *a* axis resulting in a π–π interaction with the distance Cg_1_···Cg_2_ = 3.5889 (7) Å (symmetry code: -x, -y, -z). The anions are linked together into chains by weak C—H···O interactions along the same direction. The cations are linked to the anions into a three dimensional network by N—H···O hydrogen bonds and weak C—H···O interactions (Table 1). There are O···Cl [3.1272 (10) Å] and C···O [3.1432 (14)–3.3753 (14) Å] short contacts. The crystal structure is further stabilized by C—H···π interactions (Table 1); Cg_1_, Cg_2_ and Cg_3_ are the centroids of the N2/C8–C9/C10/C15, N1/C1–C5 and C10–C15 rings, respectively.

### Experimental

The title compound was synthesized by disolving silver(I) *p*-chlorobenzenesulfonate (Chantrapromma *et al.*, 2006) (0.20 g, 0.67 mmol) in methanol (20 ml) which upon heating was added a solution of 2-[(*E*)-2-(1*H*-Indol-3-yl)ethenyl]-1-methylpyridinium iodide (Chantrapromma *et al.*, 2009) (0.24 g, 0.67 mmol) in hot methanol (30 ml). The mixture turned yellow and cloudy immediately. After stirring for 0.5 hr, the precipitate of silver iodide which formed was filtered and the filtrate was evaporated to give an orange gum. Yellow block-shaped single crystals of the title compound suitable for *x*-ray structure determination were recrystalized from methanol by slow evaporation of the solvent at room temperature after a few weeks (m.p. 457-459 K).

### Refinement

H atom attached to N was located from the difference map and refined isotropically. The remaining H atoms were placed in calculated positions with d(C-H) = 0.93 Å,  *U*_iso_(H) = 1.2*U*_eq_(C) for aromatic and CH and 0.96 Å, *U*_iso_(H) = 1.5*U*_eq_(C) for CH_3_ atoms. A rotating group model was used for the methyl groups. The highest residual electron density peak is located at 0.59 Å from S1 and the deepest hole is located at 0.65 Å from S1.

### Crystal data

**Table:**

C_16_H_15_N_2_^+^·C_6_H_4_ClO_3_S^−^	*F*(000) = 888
*M**_r_* = 426.91	*D*_x_ = 1.431 Mg m^−^^3^
Monoclinic, *P*2_1_/*c*	Melting point = 457–459 K
Hall symbol: -P 2ybc	Mo *K*α radiation, λ = 0.71073 Å
*a* = 7.4891 (1) Å	Cell parameters from 8706 reflections
*b* = 13.1650 (1) Å	θ = 1.8–35.0°
*c* = 20.3428 (2) Å	µ = 0.33 mm^−^^1^
β = 98.801 (1)°	*T* = 100 K
*V* = 1982.06 (4) Å^3^	Block, yellow
*Z* = 4	0.34 × 0.28 × 0.19 mm

### Data collection

**Table:**

Bruker APEXII CCD area-detector diffractometer	8706 independent reflections
Radiation source: sealed tube	7032 reflections with *I* > 2σ(*I*)
graphite	*R*_int_ = 0.034
φ and ω scans	θ_max_ = 35.0°, θ_min_ = 1.9°
Absorption correction: multi-scan (*SADABS*; Bruker, 2005)	*h* = −12→12
*T*_min_ = 0.899, *T*_max_ = 0.942	*k* = −21→16
39049 measured reflections	*l* = −32→32

### Refinement

**Table:**

Refinement on *F*^2^	Primary atom site location: structure-invariant direct methods
Least-squares matrix: full	Secondary atom site location: difference Fourier map
*R*[*F*^2^ > 2σ(*F*^2^)] = 0.040	Hydrogen site location: inferred from neighbouring sites
*wR*(*F*^2^) = 0.110	H atoms treated by a mixture of independent and constrained refinement
*S* = 1.05	*w* = 1/[σ^2^(*F*_o_^2^) + (0.0515*P*)^2^ + 0.614*P*] where *P* = (*F*_o_^2^ + 2*F*_c_^2^)/3
8706 reflections	(Δ/σ)_max_ = 0.001
267 parameters	Δρ_max_ = 0.88 e Å^−^^3^
0 restraints	Δρ_min_ = −0.41 e Å^−^^3^

### Special details

**Table:**

Experimental. The crystal was placed in the cold stream of an Oxford Cryosystems Cobra open-flow nitrogen cryostat (Cosier & Glazer, 1986) operating at 100.0 (1) K.
Geometry. All esds (except the esd in the dihedral angle between two l.s. planes) are estimated using the full covariance matrix. The cell esds are taken into account individually in the estimation of esds in distances, angles and torsion angles; correlations between esds in cell parameters are only used when they are defined by crystal symmetry. An approximate (isotropic) treatment of cell esds is used for estimating esds involving l.s. planes.
Refinement. Refinement of F^2^ against ALL reflections. The weighted R-factor wR and goodness of fit S are based on F^2^, conventional R-factors R are based on F, with F set to zero for negative F^2^. The threshold expression of F^2^ > 2sigma(F^2^) is used only for calculating R-factors(gt) etc. and is not relevant to the choice of reflections for refinement. R-factors based on F^2^ are statistically about twice as large as those based on F, and R- factors based on ALL data will be even larger.

### Fractional atomic coordinates and isotropic or equivalent isotropic displacement parameters (Å^2^)

**Table:**

	*x*	*y*	*z*	*U*_iso_*/*U*_eq_	
Cl1	0.55212 (4)	0.54773 (2)	0.181673 (15)	0.02519 (7)	
S1	−0.02841 (3)	0.876545 (19)	0.222068 (12)	0.01458 (6)	
O1	−0.17202 (11)	0.82466 (7)	0.24875 (5)	0.02482 (17)	
O2	−0.08774 (12)	0.92193 (7)	0.15750 (4)	0.02477 (17)	
O3	0.07291 (12)	0.94718 (7)	0.26916 (5)	0.02529 (18)	
N1	0.23969 (12)	0.71072 (7)	0.41746 (4)	0.01633 (15)	
N2	0.22567 (13)	0.29591 (8)	0.64537 (5)	0.02030 (17)	
H1N2	0.208 (2)	0.2594 (15)	0.6807 (9)	0.037 (5)*	
C1	0.21569 (15)	0.80936 (9)	0.39837 (5)	0.02025 (19)	
H1A	0.2359	0.8283	0.3561	0.024*	
C2	0.16256 (16)	0.88143 (9)	0.43974 (6)	0.0221 (2)	
H2A	0.1455	0.9485	0.4259	0.026*	
C3	0.13459 (16)	0.85187 (9)	0.50329 (6)	0.0224 (2)	
H3A	0.1008	0.8997	0.5327	0.027*	
C4	0.15718 (15)	0.75206 (9)	0.52220 (5)	0.02003 (19)	
H4A	0.1374	0.7328	0.5645	0.024*	
C5	0.20982 (14)	0.67805 (8)	0.47878 (5)	0.01632 (17)	
C6	0.23338 (15)	0.57173 (8)	0.49536 (5)	0.01806 (18)	
H6A	0.2706	0.5282	0.4641	0.022*	
C7	0.20377 (14)	0.53228 (8)	0.55435 (5)	0.01702 (17)	
H7A	0.1680	0.5777	0.5848	0.020*	
C8	0.22168 (14)	0.42820 (8)	0.57473 (5)	0.01636 (17)	
C9	0.19715 (15)	0.39707 (9)	0.63827 (5)	0.01877 (18)	
H9A	0.1657	0.4398	0.6711	0.023*	
C10	0.26815 (14)	0.25655 (8)	0.58670 (5)	0.01868 (18)	
C11	0.30803 (16)	0.15650 (9)	0.57189 (6)	0.0239 (2)	
H11A	0.3097	0.1050	0.6033	0.029*	
C12	0.34503 (17)	0.13712 (10)	0.50843 (7)	0.0270 (2)	
H12A	0.3731	0.0713	0.4969	0.032*	
C13	0.34089 (17)	0.21514 (10)	0.46122 (6)	0.0267 (2)	
H13A	0.3646	0.1996	0.4188	0.032*	
C14	0.30232 (16)	0.31472 (9)	0.47616 (5)	0.0216 (2)	
H14A	0.3001	0.3656	0.4443	0.026*	
C15	0.26664 (14)	0.33719 (8)	0.54046 (5)	0.01706 (17)	
C16	0.29792 (15)	0.63874 (9)	0.36910 (5)	0.01985 (19)	
H16A	0.4080	0.6061	0.3886	0.030*	
H16B	0.3178	0.6749	0.3299	0.030*	
H16C	0.2058	0.5884	0.3574	0.030*	
C17	0.13112 (13)	0.78168 (8)	0.20886 (5)	0.01531 (17)	
C18	0.31082 (14)	0.81082 (8)	0.20881 (5)	0.01712 (17)	
H18A	0.3442	0.8785	0.2155	0.021*	
C19	0.43933 (14)	0.73877 (8)	0.19873 (5)	0.01824 (18)	
H19A	0.5587	0.7577	0.1981	0.022*	
C20	0.38669 (15)	0.63786 (8)	0.18961 (5)	0.01780 (18)	
C21	0.20887 (15)	0.60731 (8)	0.18910 (5)	0.01905 (19)	
H21A	0.1764	0.5395	0.1826	0.023*	
C22	0.07961 (14)	0.68040 (8)	0.19856 (5)	0.01777 (18)	
H22A	−0.0403	0.6615	0.1980	0.021*	

### Atomic displacement parameters (Å^2^)

**Table:**

	*U*^11^	*U*^22^	*U*^33^	*U*^12^	*U*^13^	*U*^23^
Cl1	0.02721 (14)	0.01858 (13)	0.03119 (14)	0.00623 (10)	0.00897 (10)	0.00183 (10)
S1	0.01485 (10)	0.01459 (11)	0.01460 (10)	0.00137 (8)	0.00322 (7)	0.00272 (8)
O1	0.0196 (4)	0.0241 (4)	0.0330 (4)	−0.0001 (3)	0.0107 (3)	0.0088 (3)
O2	0.0280 (4)	0.0255 (4)	0.0211 (4)	0.0071 (3)	0.0046 (3)	0.0085 (3)
O3	0.0208 (4)	0.0233 (4)	0.0310 (4)	0.0017 (3)	0.0015 (3)	−0.0100 (3)
N1	0.0178 (4)	0.0155 (4)	0.0150 (3)	−0.0022 (3)	0.0004 (3)	0.0002 (3)
N2	0.0231 (4)	0.0187 (4)	0.0190 (4)	−0.0001 (3)	0.0028 (3)	0.0044 (3)
C1	0.0227 (5)	0.0176 (5)	0.0196 (4)	−0.0029 (4)	0.0003 (4)	0.0032 (4)
C2	0.0240 (5)	0.0156 (5)	0.0254 (5)	0.0000 (4)	0.0000 (4)	0.0021 (4)
C3	0.0244 (5)	0.0175 (5)	0.0247 (5)	0.0024 (4)	0.0019 (4)	−0.0031 (4)
C4	0.0238 (5)	0.0181 (5)	0.0182 (4)	0.0022 (4)	0.0034 (4)	−0.0008 (4)
C5	0.0177 (4)	0.0162 (4)	0.0147 (4)	−0.0002 (3)	0.0015 (3)	0.0006 (3)
C6	0.0227 (5)	0.0150 (4)	0.0169 (4)	0.0016 (4)	0.0045 (3)	0.0001 (3)
C7	0.0190 (4)	0.0159 (4)	0.0159 (4)	0.0005 (3)	0.0022 (3)	0.0000 (3)
C8	0.0179 (4)	0.0157 (4)	0.0153 (4)	0.0004 (3)	0.0019 (3)	0.0012 (3)
C9	0.0201 (4)	0.0190 (5)	0.0172 (4)	0.0010 (4)	0.0029 (3)	0.0014 (3)
C10	0.0171 (4)	0.0167 (5)	0.0217 (4)	0.0002 (3)	0.0010 (3)	0.0016 (4)
C11	0.0210 (5)	0.0156 (5)	0.0338 (6)	0.0004 (4)	0.0006 (4)	0.0016 (4)
C12	0.0237 (5)	0.0184 (5)	0.0384 (6)	0.0017 (4)	0.0032 (5)	−0.0064 (5)
C13	0.0275 (6)	0.0241 (6)	0.0290 (5)	−0.0001 (4)	0.0063 (4)	−0.0089 (4)
C14	0.0248 (5)	0.0203 (5)	0.0200 (4)	−0.0011 (4)	0.0043 (4)	−0.0030 (4)
C15	0.0168 (4)	0.0162 (5)	0.0178 (4)	0.0005 (3)	0.0015 (3)	−0.0002 (3)
C16	0.0238 (5)	0.0205 (5)	0.0155 (4)	−0.0021 (4)	0.0036 (3)	−0.0015 (3)
C17	0.0163 (4)	0.0144 (4)	0.0151 (4)	−0.0010 (3)	0.0019 (3)	0.0016 (3)
C18	0.0177 (4)	0.0146 (4)	0.0190 (4)	−0.0014 (3)	0.0027 (3)	−0.0003 (3)
C19	0.0167 (4)	0.0172 (5)	0.0208 (4)	−0.0007 (3)	0.0028 (3)	−0.0002 (3)
C20	0.0209 (4)	0.0155 (4)	0.0174 (4)	0.0029 (4)	0.0041 (3)	0.0007 (3)
C21	0.0240 (5)	0.0133 (4)	0.0205 (4)	−0.0016 (4)	0.0053 (4)	−0.0005 (3)
C22	0.0191 (4)	0.0154 (4)	0.0189 (4)	−0.0038 (3)	0.0032 (3)	0.0002 (3)

### Geometric parameters (Å, °)

**Table:**

Cl1—C20	1.7407 (11)	C8—C15	1.4515 (15)
S1—O1	1.4480 (8)	C9—H9A	0.9300
S1—O2	1.4495 (8)	C10—C11	1.3937 (16)
S1—O3	1.4615 (9)	C10—C15	1.4172 (15)
S1—C17	1.7769 (11)	C11—C12	1.3848 (19)
N1—C1	1.3595 (14)	C11—H11A	0.9300
N1—C5	1.3699 (13)	C12—C13	1.4033 (19)
N1—C16	1.4790 (14)	C12—H12A	0.9300
N2—C9	1.3531 (15)	C13—C14	1.3862 (17)
N2—C10	1.3823 (15)	C13—H13A	0.9300
N2—H1N2	0.891 (18)	C14—C15	1.4060 (15)
C1—C2	1.3673 (17)	C14—H14A	0.9300
C1—H1A	0.9300	C16—H16A	0.9600
C2—C3	1.3961 (17)	C16—H16B	0.9600
C2—H2A	0.9300	C16—H16C	0.9600
C3—C4	1.3723 (16)	C17—C22	1.3949 (15)
C3—H3A	0.9300	C17—C18	1.3996 (14)
C4—C5	1.4111 (15)	C18—C19	1.3887 (15)
C4—H4A	0.9300	C18—H18A	0.9300
C5—C6	1.4442 (15)	C19—C20	1.3901 (15)
C6—C7	1.3567 (14)	C19—H19A	0.9300
C6—H6A	0.9300	C20—C21	1.3896 (16)
C7—C8	1.4318 (15)	C21—C22	1.3990 (15)
C7—H7A	0.9300	C21—H21A	0.9300
C8—C9	1.3947 (14)	C22—H22A	0.9300
			
O1—S1—O2	113.07 (5)	C11—C10—C15	123.00 (10)
O1—S1—O3	113.29 (6)	C12—C11—C10	117.11 (11)
O2—S1—O3	112.84 (6)	C12—C11—H11A	121.4
O1—S1—C17	106.28 (5)	C10—C11—H11A	121.4
O2—S1—C17	105.82 (5)	C11—C12—C13	121.10 (11)
O3—S1—C17	104.64 (5)	C11—C12—H12A	119.5
C1—N1—C5	121.81 (9)	C13—C12—H12A	119.5
C1—N1—C16	117.49 (9)	C14—C13—C12	121.72 (11)
C5—N1—C16	120.70 (9)	C14—C13—H13A	119.1
C9—N2—C10	109.27 (9)	C12—C13—H13A	119.1
C9—N2—H1N2	125.2 (12)	C13—C14—C15	118.59 (11)
C10—N2—H1N2	125.2 (12)	C13—C14—H14A	120.7
N1—C1—C2	121.68 (10)	C15—C14—H14A	120.7
N1—C1—H1A	119.2	C14—C15—C10	118.46 (10)
C2—C1—H1A	119.2	C14—C15—C8	135.36 (10)
C1—C2—C3	118.40 (11)	C10—C15—C8	106.17 (9)
C1—C2—H2A	120.8	N1—C16—H16A	109.5
C3—C2—H2A	120.8	N1—C16—H16B	109.5
C4—C3—C2	119.79 (11)	H16A—C16—H16B	109.5
C4—C3—H3A	120.1	N1—C16—H16C	109.5
C2—C3—H3A	120.1	H16A—C16—H16C	109.5
C3—C4—C5	121.33 (10)	H16B—C16—H16C	109.5
C3—C4—H4A	119.3	C22—C17—C18	120.38 (10)
C5—C4—H4A	119.3	C22—C17—S1	121.17 (8)
N1—C5—C4	116.96 (10)	C18—C17—S1	118.44 (8)
N1—C5—C6	119.06 (9)	C19—C18—C17	120.07 (10)
C4—C5—C6	123.98 (9)	C19—C18—H18A	120.0
C7—C6—C5	123.15 (10)	C17—C18—H18A	120.0
C7—C6—H6A	118.4	C18—C19—C20	118.93 (10)
C5—C6—H6A	118.4	C18—C19—H19A	120.5
C6—C7—C8	126.99 (10)	C20—C19—H19A	120.5
C6—C7—H7A	116.5	C21—C20—C19	121.96 (10)
C8—C7—H7A	116.5	C21—C20—Cl1	119.80 (8)
C9—C8—C7	121.99 (10)	C19—C20—Cl1	118.19 (8)
C9—C8—C15	106.00 (9)	C20—C21—C22	118.85 (10)
C7—C8—C15	132.01 (9)	C20—C21—H21A	120.6
N2—C9—C8	110.32 (10)	C22—C21—H21A	120.6
N2—C9—H9A	124.8	C17—C22—C21	119.79 (10)
C8—C9—H9A	124.8	C17—C22—H22A	120.1
N2—C10—C11	128.76 (11)	C21—C22—H22A	120.1
N2—C10—C15	108.23 (10)		
			
C5—N1—C1—C2	0.81 (16)	C13—C14—C15—C10	−1.26 (16)
C16—N1—C1—C2	−179.81 (10)	C13—C14—C15—C8	−179.77 (12)
N1—C1—C2—C3	0.65 (17)	N2—C10—C15—C14	−178.58 (10)
C1—C2—C3—C4	−1.28 (17)	C11—C10—C15—C14	1.74 (16)
C2—C3—C4—C5	0.51 (18)	N2—C10—C15—C8	0.33 (12)
C1—N1—C5—C4	−1.56 (15)	C11—C10—C15—C8	−179.35 (10)
C16—N1—C5—C4	179.08 (9)	C9—C8—C15—C14	177.96 (12)
C1—N1—C5—C6	178.44 (10)	C7—C8—C15—C14	−2.8 (2)
C16—N1—C5—C6	−0.92 (14)	C9—C8—C15—C10	−0.68 (12)
C3—C4—C5—N1	0.90 (16)	C7—C8—C15—C10	178.61 (11)
C3—C4—C5—C6	−179.10 (11)	O1—S1—C17—C22	25.07 (10)
N1—C5—C6—C7	−178.80 (10)	O2—S1—C17—C22	−95.40 (9)
C4—C5—C6—C7	1.21 (17)	O3—S1—C17—C22	145.20 (9)
C5—C6—C7—C8	179.27 (10)	O1—S1—C17—C18	−155.09 (8)
C6—C7—C8—C9	176.40 (11)	O2—S1—C17—C18	84.44 (9)
C6—C7—C8—C15	−2.78 (19)	O3—S1—C17—C18	−34.96 (9)
C10—N2—C9—C8	−0.61 (13)	C22—C17—C18—C19	−0.23 (15)
C7—C8—C9—N2	−178.58 (10)	S1—C17—C18—C19	179.92 (8)
C15—C8—C9—N2	0.79 (12)	C17—C18—C19—C20	−0.80 (15)
C9—N2—C10—C11	179.81 (11)	C18—C19—C20—C21	1.17 (16)
C9—N2—C10—C15	0.15 (12)	C18—C19—C20—Cl1	−176.49 (8)
N2—C10—C11—C12	179.53 (11)	C19—C20—C21—C22	−0.48 (16)
C15—C10—C11—C12	−0.86 (17)	Cl1—C20—C21—C22	177.14 (8)
C10—C11—C12—C13	−0.48 (18)	C18—C17—C22—C21	0.93 (15)
C11—C12—C13—C14	0.92 (19)	S1—C17—C22—C21	−179.23 (8)
C12—C13—C14—C15	−0.01 (18)	C20—C21—C22—C17	−0.57 (15)

### Hydrogen-bond geometry (Å, °)

**Table:**

*D*—H···*A*	*D*—H	H···*A*	*D*···*A*	*D*—H···*A*
N2—H1N2···O1^i^	0.891 (18)	1.864 (19)	2.7541 (14)	176.2 (18)
C1—H1A···O3	0.93	2.53	3.2380 (14)	133
C7—H7A···O2^ii^	0.93	2.59	3.3067 (14)	134
C14—H14A···O2^iii^	0.93	2.52	3.2605 (14)	137
C16—H16C···O2^iii^	0.96	2.37	3.2645 (15)	156
C19—H19A···O1^iv^	0.93	2.30	3.1432 (14)	151
C21—H21A···O3^iii^	0.93	2.55	3.1885 (14)	127
C4—H4A···Cg3^v^	0.93	2.85	3.5956 (11)	138
C16—H16A···Cg1^vi^	0.96	2.72	3.4622 (12)	134
C16—H16B···Cg3	0.96	2.67	3.5533 (11)	153

Symmetry codes: (i) −*x*, −*y*+1, −*z*+1;  (ii) *x*, −*y*+3/2, *z*+1/2; (iii) −*x*, *y*−1/2, −*z*+1/2; (iv) *x*+1, *y*, *z*; (v) *x*, −*y*+1/2, *z*−1/2; (vi) −*x*+1, −*y*+1, −*z*+1.

## References

[bb1] Allen, F. H., Kennard, O., Watson, D. G., Brammer, L., Orpen, A. G. & Taylor, R. (1987). *J. Chem. Soc. Perkin Trans. 2*, pp. S1–19.

[bb2] Bruker (2005). *APEX2*, *SAINT* and *SADABS* Bruker AXS Inc., Madison, Wisconsin, USA.

[bb3] Chanawanno, K., Chantrapromma, S. & Fun, H.-K. (2008). *Acta Cryst.* E**64**, o1882–o1883. 10.1107/S1600536808027724 PMC2959251 21201095

[bb4] Chantrapromma, S., Laksana, C., Ruanwas, P. & Fun, H.-K. (2008). *Acta Cryst* E**64**, o574–o575. 10.1107/S1600536808003929 PMC2960744 21201916

[bb5] Chantrapromma, S., Jindawong, B., Fun, H.-K., Patil, P. S. & Karalai, C. (2006). *Acta Cryst* E**62**, o1802–o1804.

[bb6] Chantrapromma, S., Jansrisewangwong, P., Musor, R. & Fun, H.-K. (2009). *Acta Cryst* E**65**, o217–o218. 10.1107/S1600536808043547 PMC2968146 21581836

[bb7] Chantrapromma, S., Suwanwong, T. & Fun, H.-K. (2007). *Acta Cryst.* E**63**, o821–o823.

[bb8] Cosier, J. & Glazer, A. M. (1986). *J. Appl. Cryst.* **19**, 105–107.

[bb9] Ogawa, J., Okada, S., Glavcheva, Z. & Nakanishi, H. (2008). *J. Cryst. Growth*, **310**, 836–842.

[bb10] Sheldrick, G. M. (2008). *Acta Cryst.* A**64**, 112–122. 10.1107/S0108767307043930 18156677

[bb11] Spek, A. L. (2009). *Acta Cryst* D**65**, 148–155. 10.1107/S090744490804362X PMC2631630 19171970

[bb12] Weir, C. A. M., Hadizad, T., Beaudin, A. M. R. & Wang, Z.-Y. (2003). *Tetrahedron Lett* **44**, 4697–4700.

[bb13] Yang, Z., Wörle, M., Mutter, L., Jazbinsek, M. & Günter, P. (2007). *Cryst. Growth Des* **7**, 83–86.

